# The social construction of ‘dowry deaths’

**DOI:** 10.1016/j.socscimed.2014.07.044

**Published:** 2014-07-18

**Authors:** Jyoti Belur, Nick Tilley, Nayreen Daruwalla, Meena Kumar, Vinay Tiwari, David Osrin

**Affiliations:** a Department of Security and Crime Science, UCL, 35 Tavistock Square, London WC1H 9EZ, UK; b Society for Nutrition, Education and Health Action, Urban Health Centre, Room No. 110, Dharavi, Mumbai 400 017, India; c Lokmanya Tilak Municipal Medical College and General Hospital, Dr. Babasaheb Ambedkar Road, Sion West, Mumbai 400 022, India; d Burns, Plastic and Maxillofacial Surgery, VM Medical College and Safdarjang Hospital, New Delhi 110 029, India; e UCL Institute for Global Health, Institute of Child Health, 30 Guilford Street, London WC1N 1EH, UK

**Keywords:** India, Dowry death, Burns, Classification of death, Police, Post-mortem, Women victims

## Abstract

The classification of cause of death is real in its consequences: for the reputation of the deceased, for her family, for those who may be implicated, and for epidemiological and social research and policies and practices that may follow from it. The study reported here refers specifically to the processes involved in classifying deaths of women from burns in India. In particular, it examines the determination of ‘dowry death’, a class used in India, but not in other jurisdictions. Classification of death is situated within a framework of special legal provisions intended to protect vulnerable women from dowry-related violence and abuse. The findings are based on 33 case studies tracked in hospital in real time, and interviews with 14 physicians and 14 police officers with experience of dealing with burns cases. The formal class into which any given death is allocated is shown to result from motivated accounting processes representing the interests and resources available to the doctors, victims, victim families, the victim’s husband and his family, and ultimately, the police. These processes may lead to biases in research and to injustice in the treatment of victims and alleged offenders. Suggestions are made for methods of ameliorating the risks.

## 1. Introduction

In any jurisdiction, decisions have to be made about whether a death is natural or unnatural. If deemed unnatural, further decisions have to be made about whether it was accidental or non-accidental; and if non-accidental, whether self-inflicted or caused by a third party. In the event of death caused by a third party, decisions have to be made about whether anyone is culpable or not. Accounts of events leading up to the death are important in reconstructing the circumstances and cause, which in turn, inform the class into which it is placed. Those formally involved in classifying unnatural deaths vary by country and may include physicians, pathologists, district health officers, coroners, police officers, magistrates, public prosecutors, judges, and morticians (Brooke, 1974).

This paper deals with classification in India of young women’s deaths as a result of burns into three broad classes of unnatural death – accident, culpable homicide and suicide – supplemented by a further class, ‘dowry death’, that is available when the deceased is female and has been married for less than seven years. It contributes to the wider literature discussed below on factors affecting the classification of equivocal deaths. Dowry deaths may occur by various means, including poisoning, hanging or burning. Recognition by lawmakers that women in India have traditionally been vulnerable to dowry-related abuse by their in-laws, sometimes resulting in their death, has led to the enactment of special legal provisions to prevent such abuse and cruelty. We examine the ways in which women’s deaths by burning do or do not come to be suspected as, treated as, and formally classified as dowry deaths. When a death is classified thus, the woman’s husband or other family members are automatically considered suspect and we examine the impact of this classification on the subsequent prosecution and conviction of perpetrators.

## 2. Background and literature review

‘Dowry deaths’ comprise a unique category of deaths in India. The custom of payment of dowry by the bride’s family to the prospective bridegroom’s family is ancient and widely prevalent. One of the many explanations for it is that it is a form of compensation to the groom’s family for sheltering the woman for life (Ahmad, 2008). Other explanations include the concept of ‘*varadakshina*’: making a gift to the bridegroom to honour him. A third explanation invokes the Hindu Succession Act, which even after its amendment in 2005, confers less than equal property rights on the female child. As a result, customarily dowry is a one-time payment of ‘*streedhan*’ in lieu of her share of the family wealth at the time of her marriage (Sharma et al., 2002; Anderson, 2007). In addition, given low employment prospects and low earning capacity for women in general, dowry becomes a rational investment in the groom’s prospects and his high future earning potential (Van Willigan and Channa, 1991; Anderson, 2007). The more educated the woman, the more educated her potential partner ought to be and thus the higher the ‘price’ he commands in the negotiation process (Van Willigan and Channa, 1991) as brides compete for more desirable grooms (Anderson, 2007). What might have been, in an earlier time, a means of economically empowering a woman at the time of marriage, has metamorphosed in many cases into an instrument of exploitation of the bride’s family by the groom and/or his family.

When demands for cash, jewellery or goods remain unfulfilled in arranged marriages, or when the dowry is deemed unsatisfactory (Banerjee, 2013), the resulting tensions may lead to the husband or his extended family harassing the woman, sometimes to the extent of killing her or creating such intolerable conditions that she decides to take her own life. Such deaths are termed ‘dowry deaths’ in the Indian Penal Code (defined in section 304B). A conundrum for classification purposes is that both homicides and suicides can constitute ‘dowry deaths’ (Banerjee, 2013). Table 1 shows that the number of recorded dowry deaths has been growing very slowly, but it is unclear whether this is because the numbers have remained stable or reporting practices have remained unchanged. Nevertheless, the numbers are sufficiently high to generate concern.

Traditionally, dowry deaths (homicidal and suicidal) occurred through immolation. Indeed, even though in recent years dowry-related deaths as a result of poisoning or hanging may have been increasing, the term dowry death has become synonymous with ‘bride burning’ in popular discourse (Bedi, 2012; Varma, 2012). In this paper we focus only on dowry deaths as a result of burns.

The appropriate criminal justice response to the death of a woman from burns follows India’s Code of Criminal Procedure (CrPC), 1973. Section 174 outlines the response to suicide, homicide, accident, or death under suspicious circumstances, and is applied particularly to women within seven years of marriage. The police are to report the incident to a magistrate (who follows section 176 and is empowered to hold an inquest), and, with at least two people from the neighbourhood in attendance, to report on the appearance of the body and the apparent cause of death.

The Indian Penal Code (IPC) was amended specifically to deal with dowry-related violence, cruelty and dowry deaths in 1983. Section 498A IPC penalizes harassment (or any kind) of a woman by her marital family. Unnatural death of a woman within seven years of marriage attracts penal provisions of section 304B IPC. This section defines dowry death as the unnatural death of a woman following harassment or cruelty by her husband or his relatives in connection with a demand for dowry. In cases where a woman commits suicide, as a result of harassment (not related to dowry) from her husband or his relatives, section 306 IPC addresses abetment of suicide. If it is a dowry-related suicide both sections 304B and 306 are applicable.

Finally, amendments to the Indian Evidence Act (IEA) introduced a presumption of abetted suicide, which is a form of dowry death, and a separate presumption of dowry death (Ravikanth, 2000). Section 113A of the IEA gives the court the powers to presume abetment on the part of the husband or his relatives if a woman commits suicide within 7 years of marriage, if the husband or his relatives subjected her to cruelty. Section 113B provides that the courts ‘shall’ presume dowry death in case of unnatural death of a woman within 7 years of marriage, where prior to death either the husband or his relatives subjected the woman to harassment or cruelty. The 91st Report of the Law Commission enumerated the need for such a presumption in order to ensure that an unnatural death of a woman will entail “the need for investigation by the police or an inquest by the Magistrate into the cause of death becomes strong” (LCI, 1983: 4).

### 2.1. Classifying dowry deaths

The methods of – and difficulties entailed in – classifying unnatural deaths have been highlighted in previous research (Atkinson, 1971; Taylor, 1982). Atkinson’s (1971; 166) research highlighted the fact that “technological, cultural and administrative influences” on the production of official mortality statistics (referring to suicide statistics) were seldom acknowledged. He suggested that the study of the processes – social and legal – by which official classifications of death are produced can be useful in placing official statistics in perspective and in identifying whether any systematic, non-random biases affect their production.

We explore the process of classification of death of women by burning within the conceptual framework of ‘death brokering’ (Timmermans, 2005), which refers to activities of authorities to render individual deaths culturally appropriate. Timmermans (2005) acknowledges that ‘death brokering’ of unexpected deaths by forensic experts in late modern Western societies involves classifying “profoundly equivocal deaths into contested moral categories”. These forensic experts employ a variety of “cultural scripts” to render sense to these seemingly senseless deaths (2005: 995). In India, the police are primary ‘death brokers’ and employ culturally appropriate scripts to classify death of a woman within seven years of marriage as dowry-related (or not). This involves police officers engaging in a set of social negotiations with the victim, her natal (family of birth) and marital (husband’s family) families, health practitioners, and forensic experts to render the definition of an individual death socially and legally acceptable. How these cultural scripts come to be constructed becomes vital in understanding how dowry deaths are ‘brokered’.

### 2.2. Burning as a cause of women’s deaths in India

Fire-related injuries are the leading cause of death among women in India in the age group 15–34 years (Sanghavi et al., 2009). Public health research has focused on the causes of burn-related deaths among women, their patterns and trends (Sawhney, 1989; Sharma et al., 2002; Ambade and Godbole, 2006; Peck et al., 2008; Ahuja et al., 2009; Sanghavi et al., 2009). Collectively the research literature suggests that accidents are responsible for a majority of burns, followed by suicide attempts and finally by homicidal attempts (Sawhney, 1989; Mago et al., 2005; Ambade and Godbole, 2006; Ambade et al., 2007; Kumar et al., 2007; Ahuja et al., 2009; Chakraborty et al., 2010; Ganesamoni et al., 2010). Shaha and Mohanty’s (2006) analysis of victim and burn characteristics, based on post mortem reports, found that a majority of female victims were in the age group 18–26 years; were relatively uneducated; were from lower socio-economic positions; and were predominantly Hindu. In the cases studied burns mostly occurred during the daytime, in closed spaces, and kerosene was the main medium used.

While some epidemiological studies of deaths by burning acknowledge the possibility that misclassification of death can occur (Sanghavi et al., 2009), analyses are still conducted on the presumption that the classification can be and is unambiguous. However, the classification of a woman’s death within seven years of marriage is far from straightforward in India. It is especially hard to distinguish between some kinds of intentional and accidental deaths. Neeleman and Wessely (1997) (citing Rockett and Smith, 1995) suggest that some methods of death such as drowning (‘soft methods’) are less easily classified as definitely suicidal than others such as hanging (‘hard methods’). Extending their logic, we suggest that death by burning is a ‘soft method’ in terms of ambiguity in determining whether it was accidental or intentional. Public health studies support the suggestion that classification of death by burning is difficult (Sanghavi et al., 2009).

The legal, moral, and forensic, ambiguities involved in classifying an unnatural death suicide, as discussed in traditional psychological and sociological research (Durkheim 1897, Stengel, 1964; Douglas, 1970; Atkinson, 1971; Taylor, 1982; Pescosolido and Mendelsohn, 1986; Neeleman and Wessely, 1997; Lindquist and Gustaffsson, 2002; Shiner et al., 2009; Scourfield et al., 2010; Claassen et al., 2010), are intensified in the socio-legal context of classifying unnatural deaths of women within seven years of marriage in India, regardless of the cause of death. The literature shows that factors influencing death investigations include racial, religious, cultural, and family concerns which affect medico-legal verdicts (Carpenter et al., 2011; Huguet et al., 2010; Timmermans, 2006). This study contributes to the wider literature on death classification.

### 2.3. Formal procedures for classifying women’s deaths by burning

**Fig. 1:**
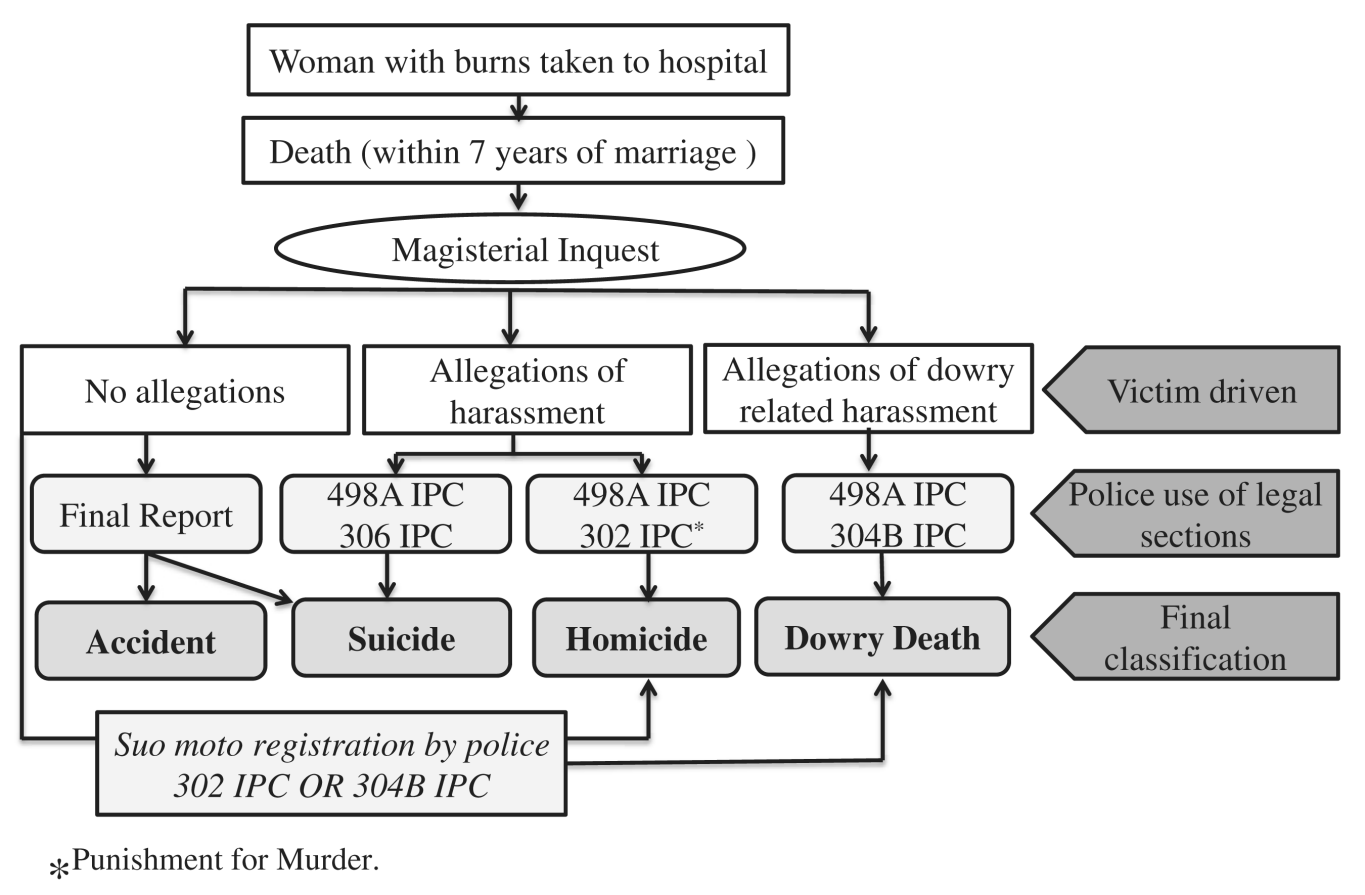
Official procedure for classification of death of women from burns.

The formal procedure for classifying the death of a woman within seven years of marriage and the available classes and relevant statutes are shown in Fig. 1.

When a woman suffers burns she is, more often than not, taken to the hospital. The police are informed by the hospital and a ‘medico-legal’ case is established. The police attend the hospital to gather available information there. The police secure the scene of the incident. A magistrate is called and records a ‘dying declaration’ (DD) that provides an authoritative victim account of the circumstances surrounding her burns. The DD may be taken by someone else, normally a doctor or police officer, if the magistrate is unable to record it, for example, because the woman fails to survive long enough. The magistrate holds an inquest, drawing on the DD, a post-mortem and statements from the women’s family. Finally, the police classify the death taking advice from findings from the magistrate’s inquest, into one of the categories shown in Fig. 1.

The inclusion of dowry deaths as a formal offence category in India reflects both an acknowledgement of and a means of trying to deal with violence against women. This is as a result of the way sexual divisions are played out in the sub-continent. Moreover, the emphasis on the accounts given by dying women themselves represents an effort to give them an authoritative voice, where they would clearly otherwise have none in bringing perpetrators to justice. The victim can act as her own witness. In the expectation that she is likely to die she may be presumed to have no vested interest in misleading the magistrate over the course of events leading to her demise. The testimony in her account is therefore granted special privilege.

## 3. Data and methods

A total of 59 semi-structured interviews were conducted with three groups of respondents: women (and their family members) admitted to two major burns units over two months (May–June) in 2012, health care providers, and police officers. The research sites were two of the largest burns units in two of India’s largest cities, Delhi and Mumbai. The researchers had working relationships with the hospital and police in Mumbai and chose the hospital in Delhi with the largest burns unit in the country as comparator.

Three research assistants were attached to the burns units in both hospitals to conduct case studies for a period of 45 days. They interviewed 33 people, including admitted women who were capable of interview (with family members in some cases), or family members of those unable to take part in an interview. In addition, 14 semi-structured interviews were undertaken with doctors, forensic pathologists and nurses by the third and last authors, and 14 with police officers by the first author. Sampling was purposive.

All interviews took place in private areas, were audio-recorded and transcribed in full. Two interviews with practitioners were conducted with two respondents together. Equal numbers of clinicians, including doctors, forensic pathologists and nurses working in the burns wards were interviewed in each city. Eight police officers in Mumbai and six in Delhi were interviewed. Officers working in five police stations in each city, where cases of women with burns had been investigated in the past year, were selected and officers of the rank of Assistant Commissioners and Inspectors with relevant investigative experience were interviewed.

Interviews were conducted in one or more languages, including English, Hindi and Marathi. All the interviews were translated literally into English and then transcribed. As a result, the quotes are sometimes in non-standard English because they are reported verbatim. Framework analysis (Ritchie and Spencer, 1994; Srivastava and Thomson, 2009) was used to examine the processes by which the police classify deaths. The analysis consisted of five steps: classifying the interview material into codes and categories; identifying a thematic framework; indexing; mapping to explore links between various themes; and interpreting and analysing emerging links from the data. The first three and last authors were extensively involved in this process.

## 4. Ethical approval

The project was approved by the Institutional Ethics Committee of Lokmanya Tilak Municipal Medical College and General Hospital (IEC/14/12), the Ethical Committee of VM Medical College and Safdarjang Hospital (58-11-EC4/8), and the UCL Research Ethics Committee (3546/002).

## 5. Findings

Respondents largely agreed on the early processes described in Fig. 1. Medico legal cases were registered in every event of burns. Police took cognizance of all serious burns patients who might die and whose deaths could potentially be classified as dowry-related (female within seven years of marriage), and every attempt was made to record their dying declaration, preferably by a Magistrate. There was no evidence that some were filtered out earlier. However, there was disagreement between the police and other interviewees over whether scenes of incidents were always visited and secured promptly.

In the following sections interviewees’ perceptions on the various strands that influence police classification are drawn together.

### 5.1. The victim’s account: her dying declaration

The DD is treated as an authoritative statement for classifying the incident along lines summarized in Fig. 1, in the event that the woman dies. The DD usually follows one of three major narratives, with obvious implications for classification:

1. My clothes caught fire in a kitchen accident.
2. I was attempting to commit suicide by setting myself on fire.
3. My husband and/or his family set me on fire.

What became clear, however, is that the woman’s account in her DD is constructed in ways shaped by the situation in which it is made and is also liable to change as her situation changes. For the 33 women involved in our case studies, 22 incidents were described by themselves or their relatives as accidental, five as suicidal, and six as homicidal. Interviewees claimed that, more often than not, in her first statement the woman usually explains her burns as a kitchen accident. Subsequently, when her parents arrive at the hospital, they encourage her (if she is still alive) to tell the truth about the incident,

Several interviewees, including eight clinicians, nine police officers and four victims or their families, mentioned that often initial accounts given by the victim change over time. As one interviewee put it:

“… there are quite a few patients who want to change their statements. Initially they come, the in-laws are involved in burning, the in-laws are with them, so initially they pretend that it was an accident. Two days later the parents of the girl arrive, then she has the guts to say, no, it was not an accident, it was this.” (SKII Doctor, Delhi).

While families sometimes provide the woman with the necessary courage to tell the truth, at other times their influence can be more pernicious. One interviewee described her experience:

“To the extent that occasionally I feel that the girl or woman who’s burned is kind of tutored by her family members to say that this was homicide. Change the statement, though it really might be suicide. Simply put the net on the in-laws.” (M3, Clinician, Mumbai).

This view was reinforced by a police officer, who interpreted the motivations behind why such changes in account might happen,

“Mostly it is like this – on the first day she had not made any allegations and then there are not very good relations between the two families and we know that the in-laws were troubling her. The girl’s parents tell her, why did you say like this, why did you not tell the truth and all that. So the girl says, ok, I will tell the truth and then the family members come and register a report saying that please record our daughter’s statement again.” (P11, Inspector, Delhi)

One factor at work in the victim’s initial account of her burns was deemed to be fear: fear of the in-laws who are around the victim at the time of admission, or fear of the future, how she would return to her marital home or who would look after her children. As time passes, other influences came into play. One physician, who disbelieved many of the initial accounts he heard, said of one woman,

“… she was almost 80 to 90 percent burnt and that time she was telling it was accidental. I sent all the relatives outside and I spoke with her *ki* [that] you don’t worry we all are there. Whatever has happened you please tell us. So that time she told *ki* ‘family members, my mother-in-law and my husband they burnt’.” (M2, Doctor, Mumbai).

A police officer said that,

“Women have a psychology that they are very emotional – maybe that is that they have emotions that why should I trouble my husband, my children need looking after, I might survive or not. So she tells the doctor no – it was a mistake, it was an accident … After a couple of days her family members meet her, she discusses this with them and then her version changes. I have seen many instances of this where the woman has changed her version thrice in three days or twice in four days. (P11, Inspector, Delhi)

Two noteworthy points are raised by the officer: first, that whether the woman will ‘tell the truth’ depends upon the relations between the two families; and second, that the woman’s family is expected to take the initiative and contact the police with a request to record her statement again.

### 5.2. Woman’s natal family accounts of cause of death

The woman’s family’s testimony is of vital importance in both the inquest and subsequent classification of death by the police. Even if she has made no accusations in her DD, but her near relatives allege harassment, officers asserted that they had no choice but to register a case against the husband or in-laws. Respondents suggested that the accounts by family members often failed accurately to report the circumstances surrounding the woman’s death. They might choose to portray it as an accident or allege dowry death, depending upon circumstances and their situation.

When asked to explain why victims or their families assert that the cause of burns was accidental when it was probably not the truth, one interviewee said,

“Her father-in-law says that. Don’t register the FIR (First Information Report) … I said why should we harass them [in-laws], we will do according to the wishes of daughter, if she says I have to go there [to her marital home], why FIR? Why should we break her home?” (R1b, Father of patient, Delhi)

Clearly, this interviewee did not report the real cause of burns as being homicidal because of pressure from his daughter’s in-laws and because his daughter wanted to return to her marital home when (and if) she recovered. Initiating criminal proceedings would jeopardise her return to her husband’s home. Another reason mentioned for reluctance on the part of the victim or her family to accuse the in-laws of homicidal intent, even if true, was concern over the upbringing of children. A consultant clinician in the burns ward in Delhi said,

“The in-laws say * “We won’t look after your child if you open your mouth.”* So because of fear the patients do not say what actually happened.” (SK1, Consultant, Delhi)

Previous research has shown that natal families use allegations of dowry death to conduct negotiations with the woman’s in-laws during the run-up to the trial to share or transfer the upbringing of the children (Berti, 2010). On the other hand, sometimes it was also the case that the victim’s family allege harassment because,

“… parents think, our daughter is now gone and he has behaved rudely with us and we don’t want to have any connection with that family, so why should we not do something to give them maximum trouble? Her family thinks like that and the police have no option.” (P4, Inspector, Mumbai).

### 5.3. Physician accounts of cause of death

Shaha and Mohanty (2006:105) found that “parents and in-laws of the deceased are becoming adept at manufacturing circumstantial evidence to serve their own interests. Therefore, to establish the truth and for the smooth administration of justice, reliable unbiased medical evidence has to be corroborated”. Health practitioners are primarily concerned with providing specialist care and treatment to women with burns on admission to hospital. Nevertheless, clinicians at the frontline in emergency or burns wards take an initial history from the patient or her relatives, register a medico-legal case and inform the police stationed in the casualty ward. This process was explained by a doctor in Mumbai:

“For example the woman coming in casualty with burns … the casualty officer goes sees the patient, if she is able to talk, asks her the name, age where she stays and how you get the burn. If she is able to tell we directly ask her and then we call the relatives and ask other details … and then we enter alleged history of so and so and so. … we ask the casualty police to see *ki* [that] this is the thing, she is saying *ki* [that] burnt by so and so person or suicidal, homicidal whatever.” (M2, Doctor, Mumbai)

Another doctor said that his involvement was restricted to taking down the history or cause of burns as narrated by the patient or relatives; he did not usually delve into the details of the incident.

“We do not go into the depth of that history … how the stove blast has occurred. They usually give the history that a stove blast has occurred, they were cooking something and then suddenly the stove blasted … But we also do not go into that much of a detail *ki* [that] whether the stove was faulty and where was she sitting and what was she wearing … in casualty area we are mainly more focused on the treatment part.” (M4, Doctor, Mumbai)

The same respondent added that, even when doctors find that the narrative they have been given and the burn patterns do not accord with one another, they seldom have the time or inclination to follow it up in order to try to ascertain the sequence of events that led up to the burns.

“But still, see, we have a lot of work over here and in that we do not get that much time. Even if we think to know *ki* what was that exactly happened, whether it was suicidal or accidental. See, residents they are working here and there and they do not take that much stress … they just ask *kya hua tha* [what happened]? Suicide, was they *likh de* [write it] … you know … it’s just … forget it.” (M4, Doctor, Mumbai).

### 5.4. Pathologist accounts of cause of death

Shaha and Mohanty’s (2006) research on dowry deaths indicated that medical evidence related only to the cause of burns (mainly kerosene) and describes the position of burns and the total body surface area (TBSA) burnt (on average, 40–80%), as well as describing accompanying characteristics such as singeing of hair and sooty blackening of skin. They found that the evidence did not discuss how burns in alleged dowry deaths are distinguishable from burns resulting from accidental causes. In the absence of clear medical evidence about the classification of death, establishing whether death was accidental or intentional becomes the responsibility of the police. Post mortems (PMs) of burns victims help establish whether there were any ante mortem injuries or poisoning, or post mortem burns, and provide some objective evidence to support allegations of dowry death.

Clinicians claimed that there were certain burn patterns associated with accidental or intentional burns and that these could be used to check the victim’s narrative. One doctor said,

“Sometimes you find very ridiculous cases where she says she was sitting and cooking. If you were sitting and cooking and it caught fire then you will be burnt from your foot upwards. You find that she is head-waist burnt and she is saying that I was sitting down and cooking. That’s not true so obviously there is some kind of pressure …” (SKII, Doctor, Delhi).

We asked police officers whether they consulted attending clinicians and pathologists while classifying the woman’s death. A mixed picture emerged. One police officer said that in some cases they had informal discussions with the attending doctor or forensic pathologist, but it was not incumbent on them to do so since they would act as ‘expert witnesses’ in the event of a subsequent trial.

“Doctors are experts, so we don’t take their statements in our system. Their PM notes are the basis on which the charge sheet is made. They come to court and say [give evidence]” (P2, Inspector, Mumbai).

Another officer said that doctors seldom gave a definite opinion on whether the burns were accidental or intentional. Since they are expert witnesses, pathologists also do not commit to any particular classification,

“No, the doctors don’t give any opinion on that. They just give what injuries were there and how these might have been caused. We have to discuss that with forensic experts. They also do not give any of this in writing, but if we discuss the matter they will say that this [suicide or dowry death] is a possibility. Then we have to infer after we inspect the scene of offence.” (P5, Inspector, Mumbai).

A forensic pathologist told us that the PM was conducted after they had read the inquest and police reports and were aware of the circumstances and alleged cause of burns:

“Once the body is brought to the mortuary, we see the inquest papers, we see the statements of the victim’s parents, relatives – brother, sister, mother, father – whoever he or she is, and then we do the post-mortem. (SKII5, Forensic Pathologist, Delhi)

The same expert went on to say,

“[If] he or she had given a dying declaration or a statement, that this and this work has been done by this and this intention, then only we can go in the direction of a homicidal intent. This is the thing as far as burns cases are concerned” (SKII5, Forensic Pathologist, Delhi).

This implies that the PM would look for relevant evidence to support (or not) the claim, only if there are allegations of homicidal intent. The risk would be that, in cases in which no allegations are made, the PM would not ‘go in that direction’. Whilst our research did not directly reveal this weakness, the possibility exists: recognizing and guarding against this shortcoming might be the pathologists’ contribution to more objective classification of death.

### 5.5. Police accounts of physical evidence

Even though officers claimed unanimously that they immediately visit the scene of occurrence and take note of all relevant physical evidence, interviews with victims revealed that this was not always the case. One interviewee, brother of a victim (Mumbai) said that her husband had set her on fire, but pressurised her to report it as an accidental stove blast upon admission to the hospital. The police had visited her in the primary health care unit to which she was initially admitted, had taken her account of a stove blast and had logged the matter as an accident. The fact that the police did not return for a couple of days to revisit the woman suggested that they had not visited the scene of occurrence (where no stove had blasted) and therefore had made no attempt to substantiate claims that the burns were the result of an accident.

Preserving the scene of the incident and gathering physical evidence was, however, seen as very helpful in reconstructing the sequence of events that led up to the burning, and thus in constructing a plausible scenario to account for the burns and the subsequent classification of death. Earlier studies on burns have found that physical evidence sometimes contradicts narratives: accounts of burns as a result of a stove burst were contradicted by the absence of a stove in the kitchen (Rao, 1997). A Delhi Inspector corroborated this, explaining that there were stark differences in scenes in cases of accidental and intentional burns:

“Both the patterns are different. If someone is cooking at the stove and if the stove explodes then all the kerosene will fly all over the place, the walls, on her head also. If someone pours kerosene on someone, then it is a bit on the lower side, so we can get an idea whether the death was because of a stove blasting or she was burnt. We can know that from the scene of crime.” (P16, Inspector, Delhi)

Officers were asked whether preserving the integrity of the scene presents special challenges, especially in crowded and densely populated urban areas. They gave conflicting views. An inspector in Mumbai (P1a) claimed that, even if the incident occurs in a crowded slum, people around are very savvy and do not interfere with the evidence. On the other hand, another Mumbai inspector (P6) claimed that, on occasion, there are attempts to clean the scene and hide physical evidence, which the police have to look out for.

### 5.6. Police constructions of dowry deaths

It might appear at first sight that classification in the case of the kinds of burns discussed in this paper should be relatively straightforward, since the women were available to give an account before they died. But it is not. The police are faced with multiple accounts from different sources, some of which, including those from the victim herself, clearly reflect the interests of those producing them. Moreover, accounts are apt to change and in some instances are inconsistent with the observed patterns of burns or the physical evidence at the scene of the incident. In resolving difficulties in the practical and consequential task of death classification, the police are not without their own interests.

A police officer said that with experience the police are able to gauge whether the pattern of burns matches the version of events.

“If a woman has died because of a stove explosion, then the position of the burns is important – where was she burnt – were the legs burnt? If she was cooking on a raised slab, then where was she burnt … ?” (P4, Inspector, Mumbai).

In practice, it appears that where no allegations are made against the in-laws, or if the woman insists that the burns were accidental, the police may choose not to visit the scene of the incident or look for corroborative evidence. Two officers described cases in which they visited the scene, suspected foul play, and then persuaded the victim or her family to register a case as a crime. One officer went further and claimed, “If we see foul play then it is our duty to register the case” (P16, Inspector, Delhi Police), implying that officers can and do register the offence *suo moto* (at their own discretion), even if neither the victim nor her family members are willing to do so. In contrast, another officer felt that “Circumstantial evidence is not decisive evidence. That may be supporting evidence, but you cannot use it to make any decisions?” (P11, Inspector, Delhi Police), implying that there must be allegations or statements to support the classification of any burning act as a crime.

Where a woman makes no allegations in her DD, but her relatives subsequently allege dowry-related harassment, one officer said that the decision tends towards registering a case even if it means going against the DD. “If the circumstances are there, we take [register] the case against DD.” (P3, Inspector, Mumbai).

A final, crucial factor that affects the process of classification of death is the police officer’s assessment of the evidence and statements. Two related factors influenced police decisions: the perception of the victim and the need to protect themselves from allegations of corruption and inefficiency (Belur et al., 2014).

In line with previous research, decision-making was influenced by the perceived ‘deserving’ nature of victim (Bynum et al., 1982). Officers sympathized with the woman and claimed that they were entirely guided by what was in her best interest. When asked how, in the event of a discrepancy between physical or forensic evidence and the accounts of the victim or her natal family, the police decide between them, one officer indicated that the approach was victim-led and therefore prioritised the narrative of the victim or her family,

“We lodge the cases and we charge-sheet [a formal document of accusation prepared and submitted by the police to the court of law] them. We will go by the statement of the victim or her relatives, blood relations, and not by the evidence” (P2b, ACP, Mumbai).

The same officer went on to explain why they chose to adopt this default position,

“… because there are a lot of agencies getting into it, women’s activists and Mahila Aayog [Women’s Commission], they will say police are involved. NGOs will come and they will say you have not done it, so it is - you invite allegations otherwise. … You have to give explanations, everybody, right from senior officers to government, everybody will keep asking you, why are you [doing this]? Then they will start finding out ulterior motives …” (P2b, ACP, Mumbai Police)

Eight of the 14 officers interviewed indicated that they had protected themselves from allegations of corruption by being entirely victim-led. While officers did not openly admit to corruption, previous research on women with burns has identified that misclassification can sometimes occur because the police might misclassify deliberately or mistakenly, the reasons ranging from corruption to bureaucracy (Kumar and Kanth, 2004; Sanghavi et al., 2009). According to Ravikanth (2000), police officers not only fall short in their duties, but are guilty of corrupt and incorrect practices in investigating dowry deaths. Our research indicated that the central role of police officers and the fact that they do have a great deal of discretion (Belur et al., 2014), can create loopholes for corrupt or inefficient practices to affect the quality of death investigation.

In practice, whether a death is deemed an accident, suicide, homicide or dowry death depends heavily on allegations in the accounts of the woman or her family, and further, if allegations of harassment are made, whether they are dowry-related. The final classification follows an inverted process. Depending primarily upon whether and what allegations are made by the victim or her relatives, the police invoke particular sections of the law, which in turn determine the legal classification of death.

It is for the Investigating Officer to make the final decision on whether the manner of death was consistent with the woman’s or her family’s story. We were told that, even when the evidence does not fit the story, officers often go along with the victim’s version of it. Thus, they could either ignore evidence that might lead to convicting the guilty, or disregard evidence that might support the alleged perpetrators’ innocence, leading to miscarriages of justice and wastage of criminal justice resources (Supreme Court of India 2013; Delhi High Court, 2010). Official records indicate that, whereas the police charged someone in 93% of dowry related cases, between 2001 and 2010 only 32% of charges resulted in conviction (Crime in India 2011; Ghosh and Choudhury, 2011). These low conviction rates might partially be explained as the result of incorrect classification (and criminal charges) in the first place.

## 6. Conclusion: summary of findings and their implications

**Fig. 2:**
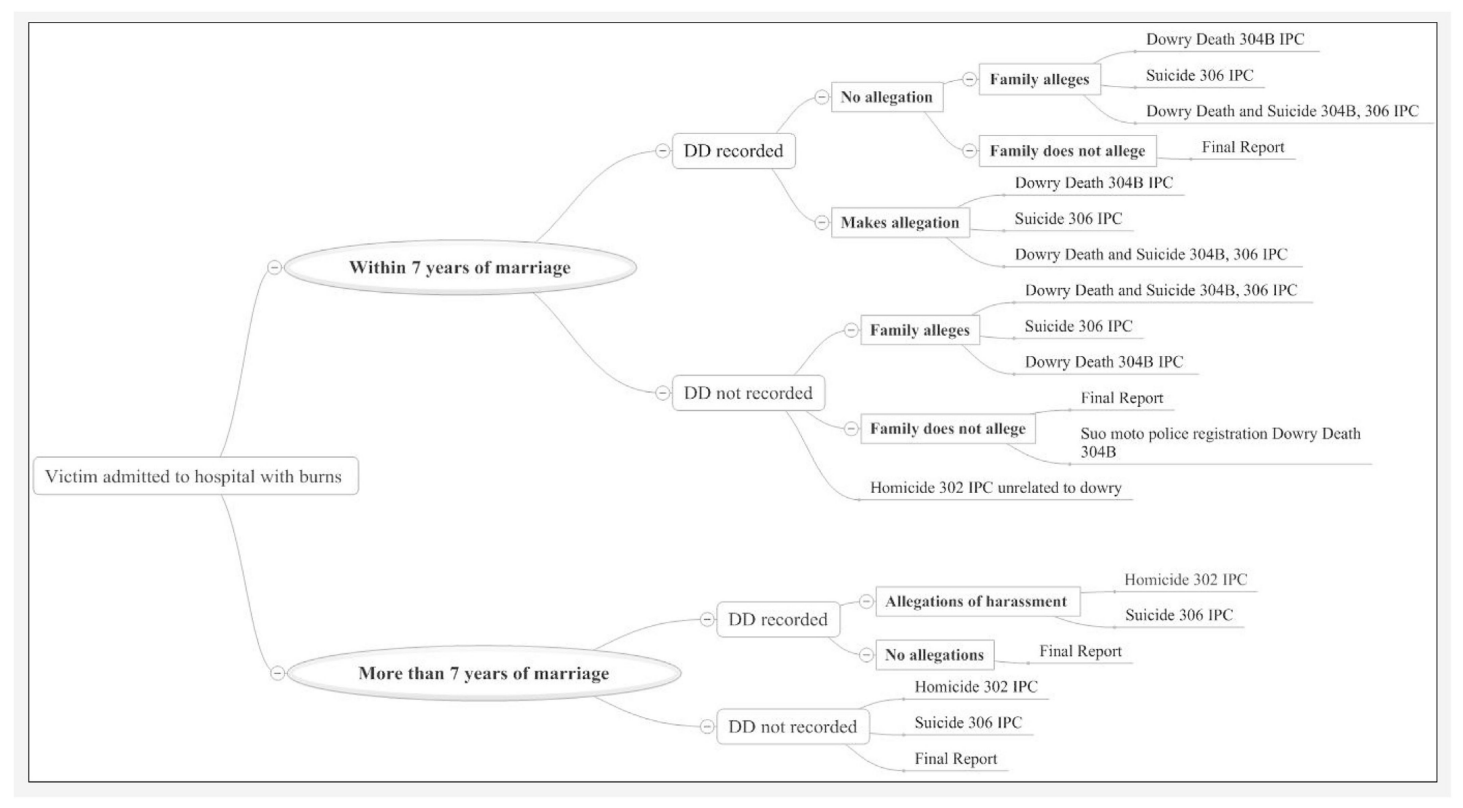
Possible pathways for classification of death of a woman as a result of burns.

This research has shown that the authoritative determination of a ‘dowry death’ follows complex social processes, with the police as the final arbiters. In this sense, the police comprise ‘death brokers’ (Timmermans, 2005). Whilst the Indian legislation relating to dowry deaths and the procedures established to identify and deal with them appear at first sight sensible and progressive, in practice we have shown that they operate in unintended ways with some perverse consequences. Fig. 2 shows the various pathways through which the death of a woman by burning may ultimately be classified.

We have shown that the accounts of deaths produced by the very women and natal families for whose protection the legislation and procedures are designed nevertheless produce situated accounts that are not mere straightforward chronicles of the events that took place. Rather, accounts are motivated by considerations that reflect the subordinate position of a married woman. Concerns about her future welfare within the husband’s family household in the event of survival, about the care of her children if she perishes, and about the injustice she may previously have endured can motivate the accounts given. These accounts are also apt to change depending on pressures and on her short-term survival expectations. What goes for the woman goes also for her family.

The police are apt to privilege uncritically the DD, sometimes in spite of contradictory evidence and often without looking to evidence that might corroborate or contradict the narrative included in it. Police decisions are situated and motivated, as are those of the dying woman. Motivations might range from corruption, protecting the victim’s interests, to protecting oneself from allegations (Belur et al., 2014). Fear of being seen to fail the dead woman, bringing the wrath of her family, and systems that tend to attach precedence to the dying declaration seem to lead systematically to failures to look for evidence that could cast doubt on the veracity of the DD. The unintended and perverse consequence can be miscarriage of justice for those wrongly accused, failure to prosecute those who are guilty of bride burning and, ultimately a reduction in the credibility of legislation designed to protect women’s interests.

The kind of evidence that could falsify, cast doubt on or corroborate the account given in the DD could come from more extensive and more routine forensic examination both of the pattern of burns experienced by the woman and the scene of the incident. Indeed, even if the woman does not in the event die, collection and analysis of forensic evidence of this kind may still provide opportunities for prosecution. Moreover, the known routine collection of independent evidence may also discourage (perfectly intelligible, but nevertheless damaging) fabrications in the accounts of events and the consequent damage they are liable to produce.

Our findings have implications for research and practice.

### 6.1. Implications for research

Systematic biases (Pescosolido and Mendelsohn, 1986) could run in both directions in relation to recorded suicides. Some ‘suicides’ may be recorded as a dowry death (hence, a homicide) in order to put pressure on the in-laws; alternatively, some homicides may be recorded as suicides in order to prevent stigma and hammer out a compromise in terms of financial compensation or if children are involved. These are not random biases and there has been little attempt to determine whether their effects, i.e. recording suicides as homicides and vice versa, cancel each other out. The research shows that social factors and social negotiations shape the accounts of burn related deaths by family members, which affects the way they are classified by the police.

These findings help put wider research on dowry deaths and classification of deaths in clearer perspective. Most research on dowry deaths is based on official statistics or hospital records, which often do not match, sometimes leading to discrepancy in findings. The findings need to be qualified by a clear grasp of the ways in which the classification of women’s deaths by burning is accomplished in practice.

The research demonstrates the need to consider cultural, technological and social factors in drawing conclusions about the spread and extent of dowry-related deaths in India (both under-reporting and over-reporting can be unacknowledged problems). Cultural factors here refer to police subcultural factors, but also wider cultural factors that influence the construction of cultural scripts which form the basis of police classification of death. We show that official statistics are an artifact of socio-cultural processes and local policing culture. Further research is needed to help understand regional variation in rates of reported dowry deaths, without assuming that the numbers accurately capture the events they are generally supposed to describe.

### 6.2. Implications for practice

The research and anecdotal evidence presented revealed that, in believing they were complying with victim wishes, police officers might respond without investigating thoroughly. When a dowry death case comes up for trial, establishing whether the cause of death was homicidal, suicidal or accidental becomes important and any slips in the investigation or original classification can mean a long delay in the judicial process or a miscarriage of justice, with the benefit of the doubt going to the accused. These are strong grounds for examining whether investigatory practices could be improved.

Sources of information that might be more extensively drawn on are those that could check the accounts given of the circumstances surrounding cases. There are at least three main possibilities: the hospital staff treating her, evidence from the scene of the incident, and the report of the pathologist conducting the postmortem. Any or all of these could be routinely used to check that the verbal accounts given are consistent with other evidence. Our findings suggest that they are currently underused. Lindquist and Gustaffsson’s (2002) research in Sweden suggests that good rapport between the police and forensic pathologists is vital in ensuring that the classification of cause of death is correct. There may be scope in India, particularly in burns cases involving young women, to make routine use of forensic experts both to check whether physical evidence is consistent with the accounts of the circumstances surrounding the burns and to suggest new lines of enquiry where inconsistencies are uncovered.

One of the solutions put forward for reducing or preventing ‘bride burning’ has been the proper implementation of existing laws, along with newer stricter legislation to abolish dowry-related crimes (Kumar and Kanth, 2004; Shaha and Mohanty, 2006). The research reported here and extensive case law has shown that the ‘victim-centred’ approach of the existing Indian dowry laws is its main strength and also its weakness. The spirit of the criminal and procedural law is intended to ensure that the woman’s (or her family’s) account is central to the classification of her subsequent unnatural death. However, as an intelligible but unintended consequence, this has compromised the letter (if not the spirit) of the law by underplaying the role of objective and forensic evidence.

We recognize that classification of the death of a woman within seven years of her marriage ought to continue to be victim-led, but recommend that the current investigative processes routinely include the collection and analysis of forensic and physical evidence to weigh the victim and family accounts that are collected.

## Figures and Tables

**Table 1:** Recorded dowry deaths in India (Crime in India 2011).

Year	2006	2007	2008	2009	2010
Number of deaths	7618	8093	8172	8383	8391
